# A Study on Cross-Media Teaching Model for College English Classroom Based on Output-Driven Hypothetical Neural Network

**DOI:** 10.1155/2022/5283439

**Published:** 2022-05-09

**Authors:** Xiangyu Guo

**Affiliations:** College of Foreign Languages, Zhengzhou University of Technology, Zhengzhou, Henan, China

## Abstract

In the field of education, the development of educational big data has become an important strategic choice to promote the construction of the digital campus and educational reform, and educational big data has become a new driving force in the field of education that cannot be ignored. Based on the theoretical basis of output-driven hypothesis neural network, and combining the media spanning of contemporary art and cross-media association effect, this study changes the status quo of English teaching through traditional methods such as grammar-translation method and deductive method and constructs a new cross-media university English teaching model. Based on the existing feature learning model of two-way attention, combined with existing techniques such as generative adversarial networks and semantic hashing, the semantic association between different media data is deeply mined, and feature learning is integrated with adversarial learning and hash learning to build a unified semantic space for different media data. In this paper, we focus on the structure and characteristics of convolutional neural networks through the study of deep learning theory, discuss three classical convolutional neural network models, such as AlexNet, VGG, and GoogLeNet, and propose a convolutional neural network model applicable to cross-media teaching in college English classroom and carry out experimental validation, and the results show that the proposed neural network model is based on output-driven hypothesis. The following research has been added to the abstract: to address the key problem of the semantic gap that is difficult to cross in cross-media semantic learning, a cross-media supervised adversarial hashing model based on two-way attentional features is proposed. Based on the existing two-way attention-based feature learning model, we combine existing techniques such as generative adversarial networks and semantic hashing to deeply explore the semantic association between different media data and integrate feature learning with adversarial learning and hashing to build a unified semantic space for different media data. The results show that the proposed neural network model of cross-media teaching in college English classrooms based on the output-driven hypothesis can not only promote the improvement of students' English literacy skills but also have a certain promotion effect on their overall performance improvement.

## 1. Introduction

With the continuous reform of English teaching and learning, the comprehensive language skills of college students have been significantly improved through the joint efforts of educational researchers and front-line teachers. Compared with traditional teaching, English teachers now pay more attention to the cultivation of output ability so that students can change the situation of “dumb English” and “unable to write” to a certain extent. The “output-driven hypothesis” has been proposed for a short period, and the current research on this hypothesis is relatively lacking, and its research objects are mostly professional college English teaching in higher education institutions. What students learn in the classroom cannot be applied in time, which is not conducive to the internalization of knowledge on the one hand and reduces students' sense of learning effects on the other hand [1]. Therefore, the current English classroom is not conducive to the improvement of students' comprehensive language skills and does not allow them to have a positive emotional experience. Motivation is a direct motivation for action; it is an internal psychological state that can motivate individuals to produce and sustain certain behaviors to achieve certain purposes. According to modern psychology, motivation has the functions of stimulation, selection, direction, maintenance, and regulation. Motivation in English learning is the source and driver of learners' English learning. In English learning, students who are motivated are more likely to develop a strong passion for learning, set high goals and face difficulties to achieve them, and continuously improve their learning level. This literacy includes four key competencies as learning ability, which is a necessary competency for lifelong learning and continuous development of students, and it puts higher demands on the cultivation of students' learning motivation [2].

Based on an output-driven hypothetical neural network, this paper clarifies the research background and significance of intelligent search of cross-media educational big data based on this algorithm and establishes a cross-media teaching model that fits the actual teaching needs of university English classrooms from three aspects: feature learning of cross-media data, deep semantic relevance learning of cross-media data, and intelligent search of cross-media data based on deep semantic learning, aiming to guide students to develop an English mindset and develop the ability to use English [3]. In this paper, a method of acquiring and preprocessing transmedia education big data is proposed, and the flowchart of the method is given to introduce the process of acquiring and preprocessing transmedia education big data in detail, including crawling, cleaning, and preprocessing of the original data. A feature learning method for cross-media education big data is proposed, i.e., a two-way attention-based feature learning algorithm that incorporates deep convolutional networks, attention mechanisms, and recurrent neural networks to learn deep semantic features of cross-media education big data, and experimental results of the feature learning method for cross-media education big data are given. The interrelationship between input and output can be seen in the proposed “output-driven-input-enabled” hypothesis. Both the output-driven hypothesis and the output hypothesis attach great importance to the role of output in ELT, but the output-driven hypothesis focuses on a more targeted population, a more explicit teaching environment, and an output approach that expands a translation ability.

This paper presents the implementation of an intelligent search system for transmedia education based on deep semantic learning. The implementation of the system is elaborated from three aspects, which are system requirement analysis, overall system design, and detailed design of system functional modules. The intelligent search system mainly contains the following functional modules: acquisition and feature learning of cross-media education big data, deep semantic relevance learning of cross-media education big data, and intelligent search module of cross-media education big data based on deep semantic learning [4]. Finally, the implementation and testing of the system are described in detail, the interface diagram of the implementation of each functional module is given, and the system testing results show that the system can realize the intelligent search of educational resource data.

The traditional topic modeling approaches are mainly latent semantic analysis, probabilistic latent semantic indexing, and latent Dirichlet assignment. One of the most classical algorithms, LDA, considers that documents are represented by a mixture of topics, and each document is a probability distribution of topics, and each topic is a probability distribution of words. However, the traditional topic models often make the boundaries between topics not easy to distinguish when dealing with short text data due to the sparsity of their according features, which in turn affects the quality of learning about topics. For this reason, many scholars have conducted relevant studies. Referring to the theory in the literature [5], there are two existing ideas to solve the problems in short textbook topic modeling: the conversion problem and the adaptation problem. Among them, the adaptation problem is to supplement and extend the short text by adding word co-occurrence to compensate for the sparsity of the short text, and the methods of adding word co-occurrence can be divided into two specific categories, i.e., learning the association between words to add word co-occurrence based on the local corpus and borrowing external semantic knowledge to improve the content of the corpus to add word co-occurrence based on the local corpus to add word co-occurrence, which is mainly to learn the semantic association between words in the local corpus to achieve global semantic associations to achieve word co-occurrence in a global sense, and the literature [6] proposes a clustering-based topic model based on the idea of clustering to cluster topics into topic clusters. Wang [7] proposes a multigranularity cluster topic model that integrates document clustering and topic modeling into a unified framework to extract local topics specific to each sequel and global topics shared across clusters. The BTM topic model proposed in the literature [8] learns topics by directly modeling the generation of word co-occurrence patterns over the entire corpus. Su et al. [9] propose a word network topic model that does not learn the topic of each document but models the topic distribution of each word, thus successfully increasing the semantic density of the data space without introducing much time and space complexity. HR, Ganesha and Aithal [10] in 2014 proposed a Dirichlet polynomial mixture model applying the Gibbs sampling algorithm, in which the DMM assumes that there is only one implied topic in each document, balancing completeness, which lies in clustering all texts into a certain cluster, and consistency, which reflects that all texts in the same cluster have high semantic similarity and is suitable for sparse and high-dimensional data scenarios. The literature [11] extended DMM using word embeddings as external extended knowledge to propose the GPU-DMM model, and thereafter proposed the improved algorithm GPU-PDMM model, both of which have good results in terms of topic consistency, text classification accuracy, and learning speed.

The “output-driven hypothesis” has received a lot of attention since its introduction, and researchers have actively applied it to various types of empirical and theoretical studies in foreign language teaching. Among the empirical studies on this pedagogy, there are more studies on higher education and university foreign language teaching but relatively few studies on high school English teaching [12]. Most of the empirical studies on the “output-driven hypothesis” in English language teaching have focused on the application of listening, speaking, reading, and writing in classroom teaching. However, the question of how motivation changes and whether there are differences in the motivation of learners at different levels of achievement has not been addressed. There are different types of motivation, and different types of motivation reflect different perceptions and attitudes toward English learning and bring about different learning experiences and outcomes, so it is important to explore the internal trends in student motivation. In the whole theoretical system of output-driven hypotheses, the teaching philosophy is oriented and is the highest goal toward which the teaching activities are directed; the teaching hypotheses provide the theoretical basis for the teaching process; the teaching process is the basic way to realize the teaching philosophy and hypotheses; the teaching process is the basic way to realize the teaching philosophy and teaching hypotheses, and at the same time, the teaching philosophy and teaching hypotheses are used as the fundamental reference in each implementation step [13].

The “key competency theory” is developed from the “whole-person education theory,” which provides more action-oriented and operable goals for English teaching. The requirements and development of key competencies are different depending on the target students, and each student has the necessary key competencies to be developed at that stage. For ordinary students, the key competencies to be developed in English learning are the same as the “core literacy” mentioned by the Ministry of Education in the English curriculum standards. The development of competence is not a quick fix, nor can it exist in isolation from the teaching of language knowledge. Teachers should integrate the development of competencies into each specific teaching activity to achieve the subtle development of these key competencies.

## 2. A Model for Teaching New Media in English Classrooms Based on Output-Driven Hypothetical Neural Networks

### 2.1. Application of Output-Driven Hypothesis in English Classroom

Any hypothesis is not a subjective assertion made out of thin air but is based on the results of previous research or established theories, and the same is true for the output-driven hypothesis. Previous research on second language acquisition has developed theories and hypotheses, including Krashen's input hypothesis and Swain's output hypothesis, among others. According to the theory of second language acquisition, the most direct means for learners to acquire information is “contact” with the target language itself, and this “contact” with the target language is called input, and when this input is processed and learned, it is called “comprehensible input.” Inspired by the “silent period” of children, Krashen argues that language can be acquired naturally with sufficiently comprehensible and adapted input that is slightly above the current level (optimal input *i* + 1), and that output is only a component, not an outcome [14]. Empirical studies have been conducted on the input hypothesis, such as the whole-body response approach, the listening and reading leadership approach, the immersion and cueing approach, and the enjoyable reading approach. At the same time, some input-output transformation models have emerged, mainly including the strategy model, the socio-educational model, the generic model, the monitoring model, the integration model of second language acquisition, and the process model of second language acquisition.

First of all, in terms of the input process, the input hypothesis emphasizes natural input and natural output, inspired by the “silent period” of children, and believes that people will have output if they have enough input. However, teaching practice proves that the emphasis on input rather than output affects the development of learners' output ability, weak output ability, more inert knowledge, and the inability to transfer declarative knowledge into procedural knowledge, which affects students' learning motivation and thus affects the quality of input. In general, the drive to rely solely on output is relatively weak. Second, in terms of the amount of input, Krashen found the input assumptions in a certain immersion French teaching program in Canada. Cross-border refers to the free matching of certain characteristics from the original resource and resources that do not seem to be connected to it. By doing so, we can add value to each other's resources and even combine them into a single autonomous entity. In China, except for some schools that can offer full English instruction, most general high schools in China have an average of ten English classes a week or about 400 minutes of study time and spend about an hour outside of class on English learning. Therefore, it is difficult to ensure the amount of input that students in general high schools need to achieve natural output. Furthermore, the teaching model proposed by the above researchers is summarized as input-output [15]. Taking reading as an example, reading strategies are determined by the purpose of reading, and different reading purposes correspond to different reading strategies, for example, reading gossip news for leisure and entertainment can choose to skip reading to see the headlines and academic papers must be read critically. For high school students, time is limited and input without purpose will be ineffective. To summarize the above questions about input assumptions, input assumptions do not guarantee when the “silent period” will end, and input must be task-driven to make input purposeful and efficient.

**Figure 1:**
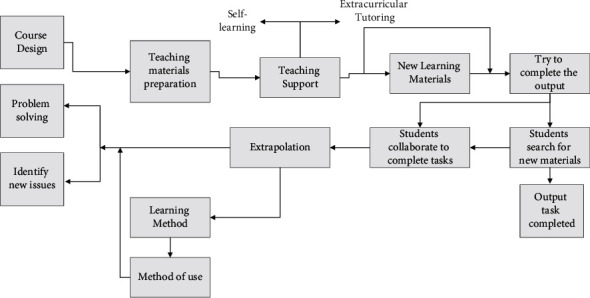
College English courses based on output-driven assumptions.

These limitations of natural input teaching make it necessary to enhance language output to improve students' English proficiency. In response to the problems of comprehensible input, the output hypothesis is proposed, which envisions that language output can make the language more fluent, make learners aware of formal deficiencies, test the output hypothesis, and promote meta-linguistic thinking. The output-driven hypothesis asserts that input is valued while output is increased. The researcher affirms several functions of output, especially the attentional and hypothesis testing functions of output. For example, Figure 1 shows the process in a college English course based on the output-driven hypothesis.

The interrelationship between input and output can be seen in the proposed “output-driven-input-enabled” hypothesis. Both the output-driven hypothesis and the output hypothesis attach great importance to the role of output in ELT, but the output-driven hypothesis focuses on a more targeted group of people, a more explicit teaching environment, and an output approach that expands a translation ability [16]. In addition, the output-driven hypothesis deepens Swain's understanding in the following aspects: firstly, the output-driven hypothesis is stronger than the input in terms of driving force; secondly, the output-driven hypothesis affirms the hypothesis of testing function in the output hypothesis and the social value of the output and believes that “speaking, writing, and translating output is more socially meaningful than reading and listening input”; finally, the output-driven hypothesis tries to verify the “social significance of output.” Finally, we tried to verify that “comprehensive output is more effective than single-item skill output.” Finally, we attempt to verify that output is more effective than input in driving second language acquisition; that the development of output skills (mainly speaking, writing, and translating) is more socially valuable than the development of input skills (mainly reading and listening); and that a comprehensive output-oriented approach to language teaching (e.g., reading, writing, listening, and speaking) is more effective than single-skill training (reading, writing, listening, and speaking) and more beneficial to students seeking employment.

This teaching theory reflects the importance of developing people who can speak, write, and translate rather than just listen and read. “The theory of teaching reflects the importance that today's society places on developing people who can speak, write, and translate rather than listen and read. The development of speaking, writing, and translating skills requires in-depth “thinking.” Writing is an advanced mode of language output and language use that reveals a person's thinking and requires students to have strong critical thinking skills [17]. Nowadays, the “text-centered” and “teacher-centered” teaching mode of high school English emphasizes input but not output, which leads teachers to focus on grammar, vocabulary, sentence structure, and essay templates in writing teaching. The teacher's input is important, but the input often makes the students' thinking ability seriously missing, and the students tend to copy and memorize rather than analyze and think deeply in the writing process. The students learn the knowledge, not necessarily the knowledge. Students do not necessarily learn the knowledge they have learned, and they do not necessarily know how to use it. The “output” is the starting point and the endpoint of the “output.” By cultivating students' thinking and understanding of the input materials and analyzing them, they can finally achieve an effective output of their ideas, which is more helpful to cultivate students with the skills of “writing.” It also helps to cultivate cognitive and discriminative talents who can analyze, reason, evaluate, and interpret the cognitive dimensions related to “writing” skills.

### 2.2. Application of Neural Network Algorithm Combining Output-Driven Hypothesis to Cross-Media Teaching in College English Classroom

Since the drive is the first part of instruction, the use of internal affective drivers to generate interest, increase student engagement, and generate positive self-evaluation of themselves contributes to the effectiveness of classroom instruction. As it is clear from the above that students' response to the drive material is one of the factors that influence their motivation to learn, optimization of the drive material becomes necessary, and therefore, the drive material should be optimized to stimulate students' internal affective drive [18]. Existing deep semantic relevance learning methods for cross-media data contain two main categories, namely, cross-media hashing algorithms and cross-media quantization algorithms. These two methods have received a lot of attention from researchers for their low storage cost and high computational efficiency. However, the existing methods, which model the data of different modalities separately, are difficult to cross the “semantic gap” between different modalities and achieve the similarity measure between cross-modal data. Therefore, reconstructing models to model the association between different modal data is a key issue for deep semantic relevance learning of cross-media educational big data. In the interviews, we found that the driving materials involve emotional factors that have some influence on students and can make them more immersed in the real situation of the driving materials. For example, some students can take the initiative to consult a dictionary for vocabulary they cannot understand when previewing the driven material. Finally, the external format is also a factor to be considered to ensure that the design of the driver materials and other formats are appealing to learners. For example, some students mentioned in the interviews that the colorful design of the materials was more willing to read.

The external input to the artificial neural network contains two kinds: the input of training samples and the desired output. This type of tutored learning approach generally requires first of all human intervention in advance to give the label of the data (desired output), and then, the artificial neural network uses the error signal between its output results and the desired output results to continuously adjust and correct each of its parameters.

(1) $$\begin{array}{c} \beta =\alpha_{1}-\frac{\left(\alpha_{2}+\alpha_{1}\right)\left(E_{n}^{1}+\lambda_{1}\right)}{\left(\lambda_{2}+\lambda_{1}\right)}, \end{array}$$

where *α* denotes the sentiment expectation of an individual and *λ* denotes the correction parameter of the neural network. The neural network is fully capable of adjusting its weights, thresholds, and even internal structure according to some specific rules or laws of the input training samples. This learning approach can be used when we face some difficult problems in English teaching and do not have sufficient experience to give the corresponding solutions. The external input to the artificial neural network contains two kinds: the input of training samples and additional evaluation information [19]. This type of learning method is different from the above two; after the artificial neural network calculates the input training samples to produce the output results, it then gives rewards (equivalent to data labels in tutor learning) and penalties through the external environment's judgment of the output results and then uses the method of reinforcing all the rewarded actions for its performance improvement and enhancement, as shown in the following equation:

(2) $$\begin{array}{c} G\left(J\right)=j\frac{\partial \gamma }{\partial j}+\frac{1}{n}\sum_{i=1}^{n}X_{i}Y_{i}. \end{array}$$

*G*(*J*) denotes the reinforcement reward option and *j* denotes the additional evaluation information. The strength of the connection between two neurons is represented by the synaptic connection weights *X*_*i*_, *Y*_*i*_, which generally take on a range between positive and negative values; if the weights are positive, the neuron is in an activated state, and if the weights are negative, the neuron is in an inhibited state. The role of the adder is to determine the total effect of each input unit, which varies in strength across neurons, and to express the total effect of the input unit on the latter neuron by their linear weighted sum [20]. The activation function responds to the relationship between the input signal and the output neuron whether it is activated or not. The neurons have different output characteristics when the activation function is different, commonly.

(3) $$\begin{array}{c} R_{k}=\sum_{i=1}^{k}f_{i}\left(w_{i}\right),\\ H\left(x\right)=\sum_{i-1}^{n}p_{i}\ln\frac{1}{p_{i}}. \end{array}$$

And continuous-type neurons generally use a nonlinear activation function, which is a continuous nondecreasing function on some closed interval, as shown in the following equation:

(4) $$\begin{array}{c} Rf\left(x\right)=\int \int g\left(t\right)\text{d}t=\left(\frac{1+\gamma }{n}\right)\cdot \sum \left(x-1\right)f\left(t\right). \end{array}$$

**Figure 2:**
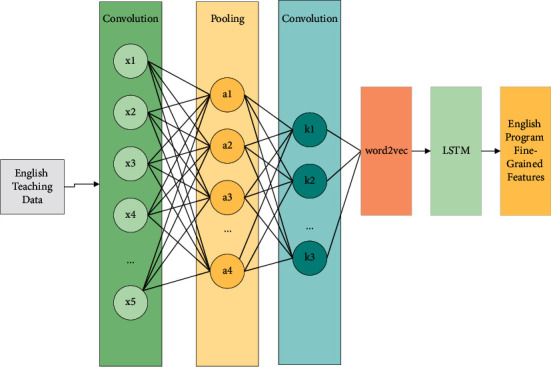
Neural network model structure of transmedia education big data.

The deep neural network structure in different modalities can also be subdivided into two parts: a fine-grained feature extraction module and a visual-semantic attention model. The fine-grained feature extraction module is used to mine the fine-grained features of a specific modality. Due to the unbalanced relationship between different modalities, the fine-grained features of different modalities cannot be accurately aligned with each other. For this reason, a visual-semantic attention model is proposed to mine the fine-grained association between two modal data and to address the problems in existing feature learning methods such as learning only global features of the data and ignoring its local feature information. In this paper, we propose a two-way attention-based feature learning model that combines a deep convolutional neural network, attention mechanism, and recurrent neural network, in which the deep convolutional neural network is used to extract fine-grained features of multimedia data, attention mechanism is used to achieve alignment between fine-grained features of multimedia data, and finally, the recurrent neural network is used to learn attention-weighted contextual associations between fine-grained features [21]. The feature learning model structure for cross-media educational big data was shown in Figure 2. The two-way attention-based neural network model, which is mainly used to learn features of raw text/image data, contains two elements, i.e., deep neural network structures for two different modalities.

Transmedia is characterized by multimodality, intertextuality, and fragmentation and also uses the intermedia association effect. Intertextuality means that transmedia publish different content through different platforms, thus accumulating fragments to help audiences gain a holistic experience [22]. Dispersion means that the audience gets occasional access from different multiple platforms to build a deeper understanding of the central idea of the story.

Cross-border refers to the free matching of certain characteristics from the original resource and resources that do not seem to be connected to it. Fully learning the complementary information and correspondence between different media data and fully mining the semantic association between different media data are the main purposes of feature learning for cross-media educational big data. To this end, the feature learning function of cross-media educational big data uses a two-way attention-based feature learning model to extract fine-grained features from two different modalities of text and image data and fully learn fine-grained associations between different modalities of data. This operation can then add value to each other's resources and even combine them into an autonomous and complete entity. From one attribute of a thing, it is possible to enter into the operation of another attribute. The subject remains the same, the attributes of the things categorized the change, and the essence is integration and fusion.

The acquisition model of cross-media educational big data and a two-way attention-based feature learning algorithm for feature learning of cross-media educational big data are proposed, and the acquisition work and feature learning work of cross-media educational big data are introduced in detail [23]. The acquisition model of cross-media educational big data is used to achieve automatic crawling, cleaning, and preprocessing of resources in the educational domain, which provides a cleaner data set for later experiments; on this basis, the two-way attention-based feature learning algorithm integrates deep convolutional networks, attention mechanisms, and recurrent neural networks in a single framework to fully learn the fine-grained features of different media data, feature alignment between them, and contextual semantics.

### 2.3. Experimental Design

**Figure 3:**
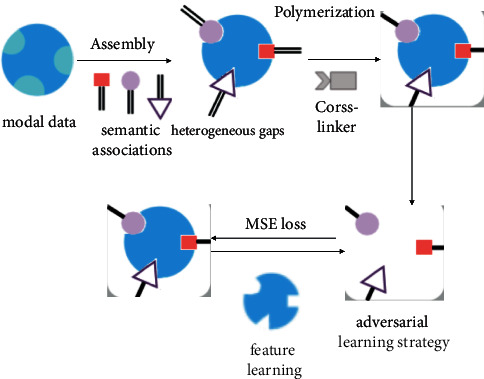
Supervised adversarial hash model based on two-way attentional features.

A supervised adversarial hashing algorithm based on two-way attentional features (as in Figure 3) is used to learn semantic associations and hash encoding across modal data. Since the distribution between different modal data is different, to solve the problem of heterogeneous gaps between different modal data, it is proposed to obtain a uniform representation between different modal data by adversarial learning strategy. Based on the generative adversarial idea, a two-way attention-based feature learning model is used as the generator *G* in the generative adversarial network, and two discriminators *D* are constructed for the data of different modalities, respectively, and the role of the discriminators is to determine whether their inputs are real data or fake data. Finally, the generators and discriminators constitute a dynamic “game process.” In addition, a hash coding network is built to hash binarize the features of the learned data with different modes. Moreover, three-loss functions are added to train the model well, including the intramodal loss function, intermodal loss function, and MSE loss function.

Since the drive is the first part of P0A instruction, using internal affective drivers to generate interest, increase student engagement, and generate positive self-evaluation of themselves contributes to classroom effectiveness. From the above, it is clear that students' response to the drive material is one of the factors that affect their motivation to learn, and the optimization of the drive material becomes necessary; therefore, the drive material should be optimized to stimulate students' internal affective drive. In the interviews, we found that the driver materials involve emotional factors that have some influence on students and can make them more immersed in the real situation of the driver materials.

## 3. Results and Analysis

### 3.1. Public Data Set Results and Analysis

To verify the effectiveness of our cross-media teaching model for college English classrooms, we set up three comparison algorithms. Specifically, first, for each label, a topic-concept space is constructed based on the embedded topic model. Then, the query vectors are classified using TextCNN, and then, three methods, Tf-ids, word2vec, and Bert, are used to calculate the semantic similarity between the query vectors and the extended words in the topic-concept space under the corresponding tags, and the candidate words that exceed the similarity threshold are selected as extended words. Weighted splicing is performed with the original query vector to serve as the final query vector.

**Figure 4:**
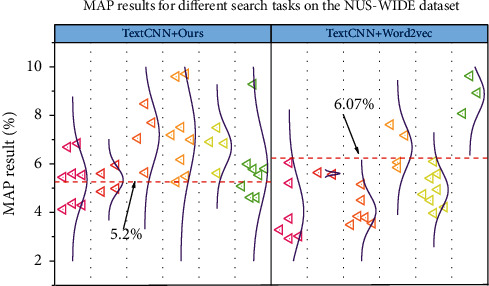
MAP results for different search tasks on the NUS-WIDE data set.

Based on the NUS-WIDE data set, the experimental results of the proposed method and the three comparison methods on MAP are shown in Figure 4. Compared with the model without semantic query expansion, the MAP values of our semantic query expansion model are improved by 5.2% and 6.07% on different search tasks, respectively. The MAP values of our semantic query extension model improved by 5.2% and 6.07% for different search tasks, respectively. And we fully investigated various word vector models, i.e., Tf-ids, word2vec, and Bert, three word embedding algorithms. Among them, Tf-ids are based on word frequency-inverse document frequency to learn statistical word vectors in the most primitive vector space, which can only learn word feature vectors based on word frequency and inverse document frequency and does not learn the semantic information of words. Word2vec has limited context and cannot solve the case of multiple meanings of words, so the semantic information of word vectors learned is also limited. Bert fully learns the context and the learned word vectors are semantically rich. Transmedia is characterized by multimodality, intertextuality, and fragmentation and also uses the intermedia association effect. Intertextuality means that transmedia publish different content through different platforms, thus accumulating fragments to help audiences gain a holistic experience. Dispersion means that the audience gets occasional access from different multiple platforms to build a deeper understanding of the central idea of the story. As the word vector model learns richer semantics, the MAP of our semantic query extension model becomes higher and higher.

**Figure 5:**
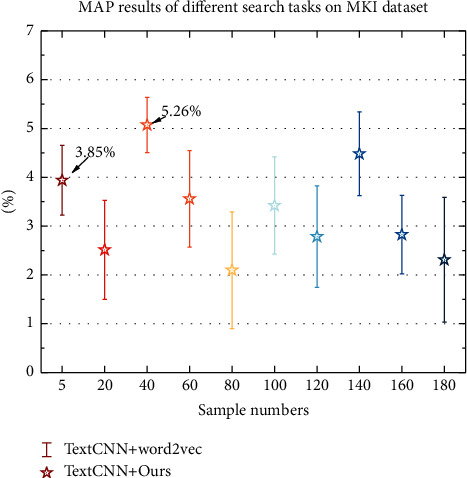
MAP results of different search tasks on the MKI data set.

Based on the MKI data set, the experimental results of the proposed method and the three comparison methods on MAP are shown in Figure 5. According to Figure 5, it can be seen that the MAP of our proposed method on different search tasks on the Mii_Flickr25K data set is significantly improved compared to that without query semantic expansion, by 3.85% and 5.26%, respectively. This is all thanks to the correct expansion and understanding of the intent of the query used through semantic expansion, which is conducive to deeper mining of the association between different modal data and thus achieving a more accurate intelligent search of cross-media data. The correct rate of target language items was 0.8288 in the experimental class and 0.7429 in the control class, with a difference of 0.0859. In terms of standard deviation, 0.1272 in the experimental class and 0.1094 in the control class, the experimental class was larger than the control class, indicating that the difference incorrect language use was greater in the experimental class. Sig (two-tailed) value of 0.004 < 0.05 and *t* value of 0.72 indicate that the experimental class was Sig (two-tailed) value 0.004 < 0.05, *t*-value 0.72, indicating that the experimental class was significantly better than the control class in the correct use of the target language, and the target language use was more grammatically correct.

Since output-driven assumes that students' output tasks are more diverse during the teaching process and the class size is large, it is a heavy task for the teacher, and it is difficult to give an appropriate assessment to each student in the classroom to ensure the learning effectiveness of each student. The main task of the facilitation session is to help students complete the output tasks by providing them with scaffolding, while the teacher provides students with the corresponding input learning materials and organizes activities that allow students to convert these learning materials from receptive knowledge to output knowledge. Therefore, in this part, the author plays the role of a guide and a facilitator, giving students appropriate guidance and help; finally, the author designs a variety of practice tasks in order from easy to difficult to make students have a deeper understanding of virtual speech and achieve the purpose of learning and using it in a live way through practicing while learning. Therefore, in addition to the public data set, a series of comparative experiments are conducted for the education domain data set.

### 3.2. Experimental Results and Analysis of the Education Domain Data Set

To fully validate the effectiveness of our proposed algorithm, experiments are conducted not only on the public data set but in addition, to on the education domain data set, for the proposed algorithm. Based on the education domain data set, our query semantic extensions on different search tasks are helpful to improve the MAP performance. Moreover, the experimental results show that our model outperforms the other comparison methods. Compared with the optimal comparison algorithm, the MAP metrics of our model are improved by 7.6% and 1.4% on the two search tasks, respectively. Compared with the algorithm without semantic expansion, the MAP metrics of our model, on the two search tasks, improve by 8.9% and 12.3%, respectively. This is mainly because the semantics of the word vector learned by Bert is richer, and it is more reliable when using this word vector to calculate the similarity with other candidates.

**Figure 6:**
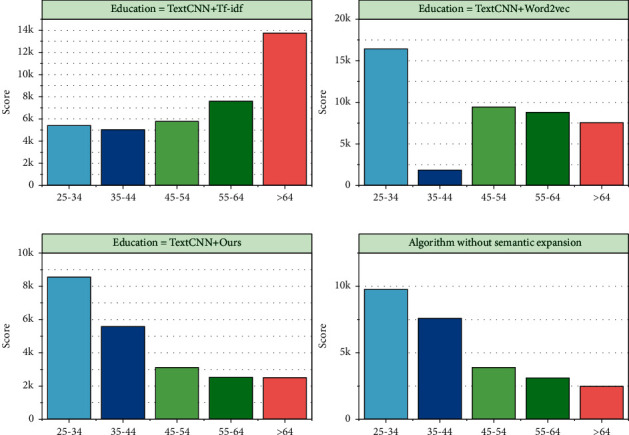
Top-*k* curve for the text search image task on the education domain data set.

The top-*k* curves for different search tasks on the education domain data set are shown in Figure 6. According to the curves in the figure, we can see that the accuracy rate gradually decreases as the value of *k* increases. This also indirectly indicates that the smaller the value of *k* in the results of top-*k* returned by our model, the higher the proportion of true examples returned. In the whole theoretical system of output-driven hypothesis, teaching philosophy is oriented and is the highest goal toward which teaching activities are directed; the teaching hypothesis provides the theoretical basis for the teaching process; the teaching process is the basic way to realize teaching philosophy and hypothesis; teaching process is the basic way to realize teaching philosophy and teaching hypothesis; and at the same time, teaching philosophy and teaching hypothesis should be used as the fundamental reference in each implementation step. Since the existing web data contain a variety of media data such as text and images, the “heterogeneous gap” problem leads to inconsistent data representations in different media, which makes it difficult to measure the similarity directly. Most of the existing feature learning methods only considered the feature learning of single media data itself. The semantic information among multimedia data with the same semantic meaning is consistent and complementary. Therefore, fully learning the complementary information and correspondence between different media data and fully exploring the semantic association between different media data are the main purposes of feature learning for cross-media educational big data. To this end, the feature learning function of cross-media educational big data adopts a two-way attention-based feature learning model to extract fine-grained features from two different modalities of text and image data and fully learn the fine-grained associations between different modalities of data.

**Figure 7:**
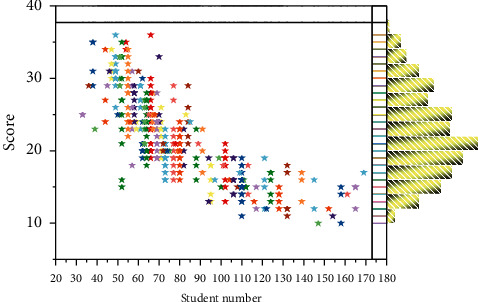
Target language item correctness rate.

Figure 7 shows the correctness of the target language items, with a difference of 0.0859 between 0.8288 in the experimental class and 0.7429 in the control class. In terms of standard deviation, 0.1272 in the experimental class and 0.1094 in the control class, the experimental class was larger than the control class, indicating that the difference incorrect language use was greater in the experimental class. The Sig (two-tailed) value of 0.004 < 0.05 and the *t*-value of 0.72 indicate that the experimental class was significantly better than the control class in terms of correct use of the target language, and the target language use was more grammatically correct. Existing deep semantic relevance learning methods for cross-media data contain two main categories, namely, cross-media hashing algorithms and cross-media quantization algorithms. These two methods have received a lot of attention from researchers for their low storage cost and high computational efficiency. However, the existing methods model the data of different modalities separately, which is difficult to cross the “semantic gap” between the data of different modalities and achieve the similarity measure between the data of different modalities. To this end, the deep semantic relevance learning function for cross-media educational big data uses a supervised adversarial hash model based on two-way attention features, which combines semantic hash coding techniques and generative adversarial ideas to fully learn the associations between different modal data based on the learned two-way attention features, construct a unified representation space between different modal data, and learn a unified common representation for different modal data.

**Figure 8:**
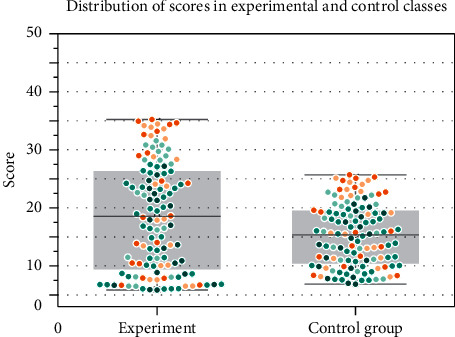
Distribution of scores in experimental and control classes.

To quantitatively clarify the differences in the scores of the 2 classes, the ANOVA results were further analyzed using the independent samples *t*-test of the SPSS statistical analysis tool to analyze the total scores, subjective scores, and essay scores of the 2 classes separately. ANOVA results showed that there were no significant differences between the total English scores, subjective scores, and writing scores of the two classes at the 0.05 level of significance, indicating that the English levels of the two classes were comparable. Combined with the score distributions of the two classes analyzed previously, the two classes met the basic conditions required for the experiment. The scores of subjective questions in the experimental class and the control class were 20.6 and 18.9, respectively, and the standard deviation of the scores of subjective questions in the experimental class was smaller than that in the control class, indicating that the experimental class had slightly better scores than the control class, and the distribution was less variable. The distribution of scores of the two classes is shown in Figure 8, and the distribution of both subjective questions and writing in the experimental class is more concentrated than that in the control class, indicating that the English subjective questions and writing in the experimental class have improved.

The above analysis shows that students with different language bases have lower English scores (total, subjective, and essay scores) than those with better scores in the experiment in general, which shows a wider distribution of scores in the class, but the test results after the completion of the experiment show that students' total, subjective, and essay scores in English have improved in different magnitudes, which indicates that the output-driven model proposed in this paper based on The hypothesis-based cross-media teaching model of neural network college English classroom can not only promote the improvement of students' English literacy skills but also have a certain contribution to their overall performance improvement.

## 4. Conclusion

The output-driven hypothesis no longer follows the previous method of teaching input before output but closely integrates output with input, which allows students to input and output at the same time and serves to promote learning. Therefore, teachers can actively use this method in their daily teaching to help students learn better. Based on the output-driven hypothesis, this paper investigates the cross-media teaching mode in college English classroom through a neural network algorithm and divides the use of cross-media educational big data to fully explore the information between multimedia data and realize the intelligent search of cross-media educational big data based on deep semantic learning, which has important research value and application scenarios. The effective effect is to create positive psychological dispositions and learning attitudes in learners and to ensure that students actively participate, learn consciously, and use active learning strategies. For example, some students can actively consult a dictionary for vocabulary that they cannot understand when they preview the driving material. Finally, the external format is also a factor to be considered to ensure that the design of the drive materials and other formats are appealing to learners. To address the key problem of the semantic gap that is difficult to cross in current cross-media semantic learning, a cross-media-supervised adversarial hash model based on two-way attention features is proposed. Based on the existing two-way attention-based feature learning model, we combine existing techniques such as generative adversarial networks and semantic hashing to deeply explore the semantic association between different media data and integrate feature learning with adversarial learning and hashing to build a unified semantic space for different media data. The experimental results show that the proposed method not only has significant performance improvement on the public data set but also has significant performance improvement on the constructed data set in the education domain, which not only reduces teachers' workload by about 15% but also improves students' learning efficiency by about 13% compared with the traditional education model.

The current phase of this paper still has some shortcomings, and in the future work, the following aspects will be studied. The feature extraction methods used in this paper, such as grammatical error determination and spelling errors, use third-party tools, which are mostly extracted in a rule-based manner, and thus cannot effectively detect syntactic and grammatical errors in complex and variable language representations. Therefore grammar, spell checking, and other tasks will be studied next. This thesis proposes the idea of model migration for ELT content topic segmentation, and the effectiveness of the idea of model migration is verified by experiments. Future work will verify the performance of model migration in the calculation of coherence scores of ELT content.

## Data Availability

The data used to support the findings of this study are available from the corresponding author upon request.
